# A Micro Capacitive Humidity Sensor Based on Al-Mo Electrodes and Polyimide Film

**DOI:** 10.3390/polym16131916

**Published:** 2024-07-05

**Authors:** Wenhe Zhou, Jiafeng Wei, Liangbi Wang

**Affiliations:** 1 School of Environmental & Municipal Engineering, Lanzhou Jiaotong University, Lanzhou 730070, China; 11220114@stu.lzjtu.edu.cn; 2 Key Lab. of Railway Vehicle Thermal Engineering of Ministry of Education, Lanzhou 730070, China; lbwang@mail.lzjtu.cn; 3 School of Mechatronic Engineering, Lanzhou Jiaotong University, Lanzhou 730070, China

**Keywords:** polyimide, humidity sensor, test system, capacitive sensor

## Abstract

Quickly sensing humidity changes is required in some fields, such as in fuel cell vehicles. The micro humidity sensor used for the relative humidity (RH) measurement with fast response characteristics, and its numerical model and method are rare. This paper firstly presents a numerical model and method for a parallel plate capacitor and a numerical analysis of its dynamic characteristics. The fabrication of this sensor was carried out based on the numerical results, and, the main characteristics of its moisture-sensitive element are shown. This parallel plate capacitor is made using complementary metal-oxide semiconductor (CMOS)-compatible technology, with a P-type monocrystalline silicon wafer used as the substrate, a thin polyimide film (PI) between the upper grid electrode and the lower parallel plate electrode, and electrodes with a molybdenum–aluminum bilayer structure. The shape of the micro sensor is square with 3 mm on the side of the source field. The humidity sensor has a linearity of 0.9965, hysteresis at 7.408% RH, and a sensitivity of 0.4264 pF/%RH. The sensor displays an average adsorption time of 1 s and a minimum adsorption time of 850 ms when the relative humidity increases from 33.2% RH to 75.8% RH. The sensor demonstrates very good stability during a 240 h test in a 25 °C environment. The numerical model and method provided by this study are very useful for predicting the performance of a parallel plate capacitor.

## 1. Introduction

Micro humidity sensors are widely used in various fields, such as in medicine, defense, transportation, environment, meteorology, industry, agriculture, storage, etc. [1,2]. Sometimes, the dynamic characteristics of the sensor are paid special attention, such as the humidity monitor for fuel cell vehicles and for the diagnosis of respiratory diseases. The sensitivity of a conductive humidity sensor is good, but it is prone to being degraded by contaminations, and its large temperature dependence is another problem [3,4,5]. Although its sensitivity is lower than that of the resistive sensor, the capacitive humidity sensor is considered a promising in some applications, especially in fields needing a quick humidity measurement. The performance prediction of a humidity sensor using a numerical method could be more comprehensive and provide a guide for the following work. Therefore, to look for an effective model and convenient method, research on a capacitive humidity sensor with better dynamic characteristics is needed.

Many studies on humidity sensors have been conducted in recent years, of which several reported achievements regarding the dynamic characteristics of humidity sensors. Compared to other interdigitated electrode capacitors with SiO_2_, polyimide, and etched polyimide, He Yu [6] developed a sensor with a sensitivity of 0.94 pF/%RH with IPD technology using polyacrylonitrile (PAN) as the additive and rutile TiO_2_ as the doping element, and the hysteresis was reduced to 0.95% RH. Guo et al. [7] reported a humidity sensor with a sensitivity of 2.2797 pF/%RH and a wet hysteresis of 2.8% RH by blending soluble polyimide with silica nanoparticles. By using multiple polyimide columns with diameters of a few microns, Uksong et al. [8] introduced a column capacitor integrated with a polysilicon heater, whose response time and sensitivity were 1.0 s and 30.0 pF/%RH, respectively. Yuta et al. [9] successfully prepared a highly sensitive polyimide humidity sensor by adopting a double layer structure of ultra-thin anodic Ta_2_O_5_ (50 nm) and polyimide (50 nm), whose response time was improved to less than 1 s by depositing a semitransparent Au top electrode and increasing the fluorine content of polyimide, and the capacitance changed almost linearly over 0–80% RH. Petr et al. [10] described a durable micro-fabricated humidity sensor made of interdigitated rhodium electrodes and a cover sensing film of Nafion perfluorosulfonate ionomer, which was the sensor with the fastest response characteristics reported to date and excellent long-term response stability even with DC excitation due to rhodium electrode, but depositing rhodium on the micro sensors was difficult. Pouria et al. [11] and AmirHossein et al. [12] reported an ultra-sensitive biomedical sensor with an average 3.923 THz/RIU based on a graphene resonator, but it requires considerable development before it can be used for environmental humidity measurements.

As sophisticated and costly fabrication technology, a gap remains between research and application for the sensors mentioned above, and numerical studies on humidity sensors are scarce. In this study, a numerical model and method as well as performance prediction on a parallel plate capacitive humidity sensor are provided firstly, and, then, the fabrication of this sensor based on Al-Mo electrodes, polyimide film, and Si substrate is introduced. Then, some characteristics are shown. The results will be valuable for the application of a humidity sensor characterized by a fast response time, high sensitivity, linearity, and stability, with fabrication being simple and cheap.

## 2. Numerical Study

### 2.1. Physical Model

**Figure 1:**
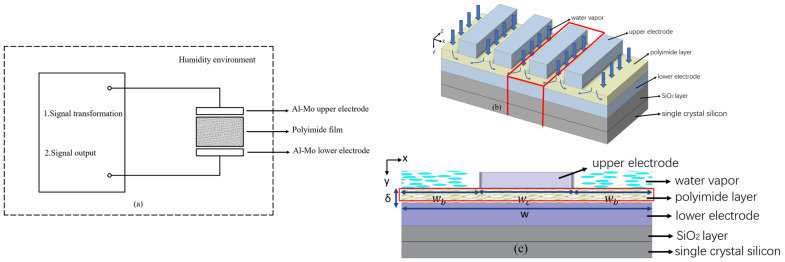
Principle of sensing humidity: (**a**) equivalent sensor circuit, (**b**) element diagram, (**c**) physical model.

Figure 1a shows an equivalent sensor circuit, and its parallel plate capacitive humidity-sensitive element shown in Figure 1b consists of a P-Si substrate packed with a SiO_2_ insulation layer and a sensing moisture film with porosity between the lower and upper electrodes. In order to allow water molecules into or out of the film easily, the upper electrode has a grid pattern consisting of bars and gaps. The dielectric constant of the dry hygroscopic film (about 2.9) is very small compared with that of water (78.54 at 25 °C). When the water molecules diffuse into the film through the upper electrodes, the dielectric constant of the film changes with the water molecule concentration absorbed into the film, which results in the capacity change of the sensor. By comparing the capacity result with the pre-calibration capacity value of the environmental humidity, the relative humidity of the object being tested can be determined.

Because the humidity-sensitive element can be considered as a combination of many of the same units, in order to save computer resources, one unit film surrounded by the red box shown in Figure 1c was selected as the physical model, whose thickness (*δ*), diffusivity (*D*), and porosity (*η*) were 0.54 μm, 2.75 μm^2^/s, and 15%, respectively. With the help of Fluent 2022 software, the diffusion process of the water molecules in the unit film was determined firstly, and then the capacitor performance was obtained by summing these units. The numerical prediction on the humidity sensor was highlighted in this research.

### 2.2. Numerical Model and Method

The apparent dielectric constant *ε_s_* of the film can be given by Looyenga’s empirical equation, shown as Equation (1) [13], and the *ε_w_* of water is given in Equation (2) [13], which is about 78.54 at 25 °C. The resulting capacitance can be described by Equation (3) [14].

(1) $$\varepsilon_{s}={\left[\gamma \left(\varepsilon_{w}^{1/3}-\varepsilon_{p}^{1/3}\right)+\varepsilon_{p}^{1/3}\right]}^{3}$$

(2) $$\varepsilon_{w}=78.54\{1-4.6\times 10^{-4}\left(T-298\right)+8.8\times 10^{-6}{\left(T-298\right)}^{2}\}$$

(3) $$C_{s}=A\frac{\varepsilon_{0}\varepsilon_{s}}{d}$$

where *ε_p_* and *ε*_0_ are the dielectric constant of hygroscopic film in a dry state, about 2.9, and the vacuum dielectric constant; *T* is the temperature in K; *A* is the film area covered by the upper electrodes, at 0.38 mm^2^; and *δ* is the distance between the two electrodes. *γ* is the fractional volume of water molecules in the film, based on the following equations [13],

(4) $$\gamma =\gamma_{m}\phi (T)RH^{\psi (T)}$$

(5) $$\phi (T)=1-\alpha_{0}(T-298)$$

(6) $$\psi (T)=\psi_{0}\frac{1-\alpha_{1}(T-298)+\alpha_{2}{\left(T-298\right)}^{2}}{1+\beta_{1}\exp[\beta_{2}(T-298)]}$$

where ϕ(*T*) [13] represents the temperature dependence of the adsorption coefficient, and *ψ*(*T*) [13] reflects the temperature dependence of *ε_w_* and catalysis with the following parameters: *γ_m_* = 4.04 × 10^−2^, *α*_0_ = 2.43 × 10^−3^, *β*_0_ = 0.836, *α*_1_ = 2.22 × 10^−4^, *α*_2_ = 2.34 × 10^−5^, *β*_1_ = 4.9 × 10^−3^, and *β*_2_ =−0.12.

If neglecting the effects of surface diffusion resistance, chemical adsorption, and the condensation of water molecules, and considering the symmetry along the *z* direction, the diffusion process of water molecules in the film can be simplified as a 2-dimensional physical process, which can be described by Fick’s second law (7) [15]. The effective diffusion coefficient of water vapor in the film *D^e^* is defined in Equation (8) [15].

(7) $$\frac{\partial N}{\partial t}=\frac{\partial }{\partial x}(D^{e}\frac{\partial N}{\partial x})+\frac{\partial }{\partial y}(D^{e}\frac{\partial N}{\partial y})$$

(8) $$D^{e}=D\frac{\eta }{\tau }$$

where *t* is time, *D* is the pure diffusion coefficient of the binary fluid in m^2^/s, *η* is the film porosity, and *τ* is the tortuosity factor of the pore structure; here, *τ* = 1 + 0.41(1/*η*).

When *t* = 0, in the region of *0* < *y* < *δ* and *0* < *x* < *w*, the water molecules’ concentration *N* equals *N*_1_, as described in Equation (9) [15], in which *φ* is the ambient relative humidity in % RH, *P*_s_ and *B* are the saturated water vapor pressure and the atmospheric pressure at the corresponding ambient temperature, Pa.

(9) $$N=N_{1}=0.622\frac{\varphi_{1}P_{s}}{B-\varphi P_{s}}$$

When *t* > 0, in the wall surface and the symmetry surface of *x* = 0, *x* = *w*, *y* = *δ*, *y* = 0, and *w*_b_ < *x* < *w*_b_ + *w*_c_, the concentration gradients are all set to zero. When *y* = 0 and *0* < *x* < *w*_b_, and *y* = 0 and *w*_b_ + *w_c_* < *x* < *w*, the water molecules’ concentration *N* equals *N*_2_, as described in Equation (10) [15].

(10) $$N=N_{2}=0.622\frac{\varphi_{2}P_{s}}{B-\varphi P_{s}}$$

Some parameters included in Equations (1)–(10) and their values are provided in Table 1.

### 2.3. Mesh and Its Independence Assessment

The response time is defined as the time spent by the sensors to sense a humidity change when the environmental humidity *RH*_1_ transiently changes to another humidity, *RH*_2_, under the same temperature. For example, *t*_30_ is the time spent when the capacitance changes from *C*_1_ to *C*_30_ of the sensor to sense humidity when the environment humidity changes from *RH*_1_ to *RH*_2_, and *C*_30_ is the sensor capacitance corresponding to the humidity of *RH*_1_ + 0.3(*RH*_2_ − *RH*_1_).

In this study, a uniform grid system was employed. Three mesh sizes of 0.018 μm, 0.02 μm, and 0.023 μm were selected to assess the mesh independence. In the calculation, the upper electrode grids and gaps were 2.0 μm/4.0 μm. When the humidity abruptly changed from 33.2% RH to 75.8% RH in an environment of 298 K and 101,325 Pa, the corresponding response times were as listed in Table 2. Based on the independence assessment, the mesh with a size of 0.023 μm was chosen for the following numerical calculation.

### 2.4. Numerical Results and Analysis

#### 2.4.1. Diffusion Process of Water Molecules

**Figure 2:**
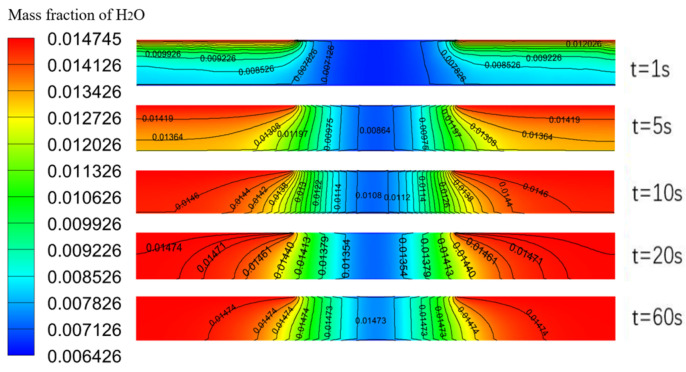
Diffusion process in the film.

Figure 2 shows the diffusion results of the water molecules in the film with time. When the relative humidity changed from 33.2% RH to 75.8% RH, the water molecules diffused through the upper electrodes into the film along the *y* direction firstly, and this process occurred transiently, because the film was very thin, and the concentration difference of the water molecules was large. And then, the water molecules diffused along the *x* direction into the region covered by the bars of the upper electrode, and this process was slow because the transport path of the water molecules was longer. The result was the opposite during the desorption process.

The results indicated that the response characteristics of the humidity sensor depended on the diffusion time of the water molecules in the film. By using a sensing film with a large diffusivity and decreasing the diffusion distance of the water molecules, the dynamic characteristics and the sensitivity of the humidity sensor could be effectively improved. The results also demonstrated the rationality of the numerical model and methods used in this study.

#### 2.4.2. Porosity Effects on the Response Time

**Figure 3:**
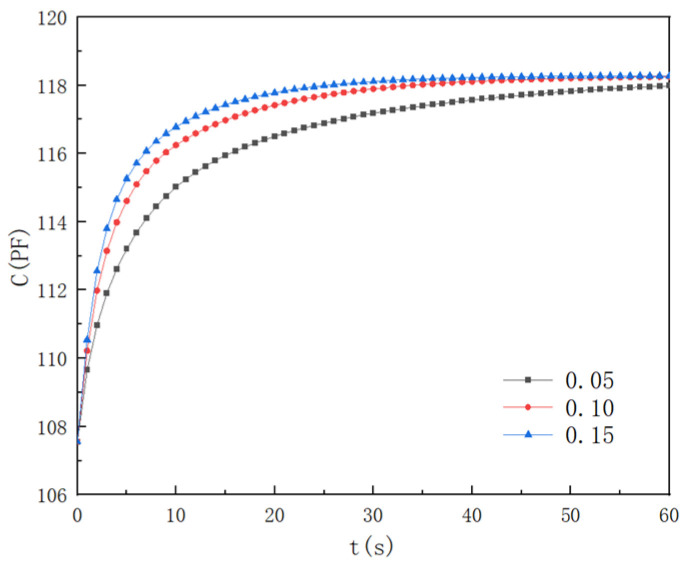
Porosity effects.

If the diffusion process of water molecules in a moisture-sensitive membrane is regarded as a purely physical process, one of the important factors affecting its diffusion is the diffusion coefficient of the moisture-sensitive membrane. According to Equation (8), the effective diffusion coefficient is closely related to the porosity of a moisture-sensitive membrane, and the larger the porosity, the larger the effective diffusion coefficient of the water molecules in the membrane, which leads to better response performance of the moisture-sensitive element. In order to indicate the porosity effects on the diffusion process of the water molecules in the films, the films with three porosities of 5%, 10%, and 15% were selected, and their corresponding effective diffusion coefficients were 1.49 × 10^−14^ m^2^/s, 5.39 × 10^−14^ m^2^/s, and 1.10 × 10^−13^ m^2^/s, respectively. When the environmental humidity changed from 33.2% to 75.8%, the numerical results of the sensor response time were as presented in Figure 3.

As the film porosity increased, the effective diffusion coefficient of the water molecules in the film and the response time of the sensor significantly improved. When the film porosity was 5%, the sensor response times of *t*_30_, *t*_60_, and *t*_90_ were 1.84 s, 6.72 s, and 30.56 s, respectively. When the film porosity increased from 5% to 10%, the response times of *t*_30_, *t*_60_, and *t*_90_ shortened by approximately 29%, 36%, and 43%, respectively. When the film porosity increased from 10% to 15%, the sensor response times of *t*_30_, *t*_60_, and *t*_90_ decreased by about 15%, 20%, and 26%, respectively. This indicated that the porosity effect on the dynamic characteristics weakened when the porosity increased. This result was verified by the following test results provided in this paper.

#### 2.4.3. Film Thickness Effects on the Response Time

**Figure 4:**
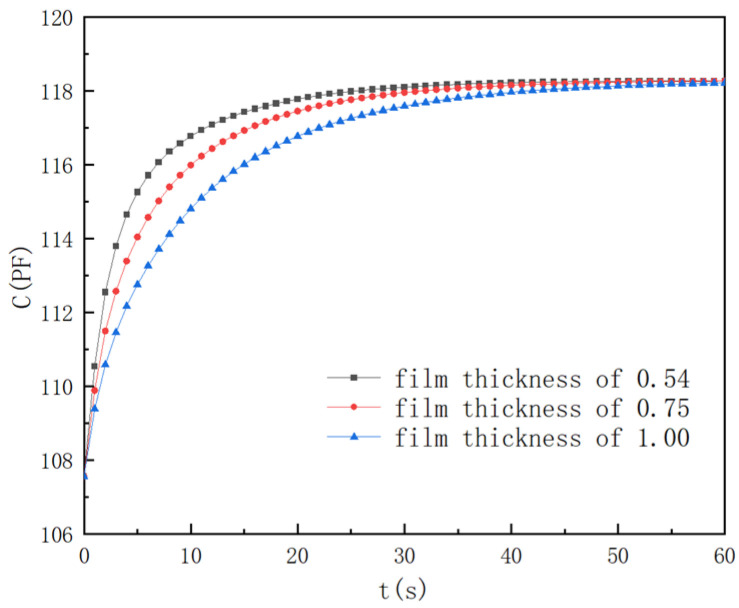
Film thickness effects.

In order to demonstrate the film thickness impact on the dynamic characteristics of the humidity sensor, sensors with film thicknesses of 0.54 μm, 0.75 μm, and 1.0 μm were selected. The other parameters were the same as above. Figure 4 illustrates the numerical results.

When the film thickness was 1.0 μm, the sensor response times of *t*_30_, *t*_60_, and *t*_90_ were 2.21 s, 7.64 s, and 24.3 s, respectively. When the film thickness decreased from 1.0 μm to 0.75 μm, the corresponding response times of *t*_30_, *t*_60_, and *t*_90_ improved by 29.9%, 35.9%, and 28.9%, respectively. When the film thickness decreased from 0.75 μm to 0.54 μm, the corresponding response times of *t*_30_, *t*_60_, and *t*_90_ improved by 28.4%, 34.5%, and 25.2%, respectively. The smaller membrane thickness helped to shorten the diffusion distance of the water molecules, thereby accelerating its diffusion process in the film.

#### 2.4.4. Upper Electrode Size Effects on the Response Time

**Figure 5:**
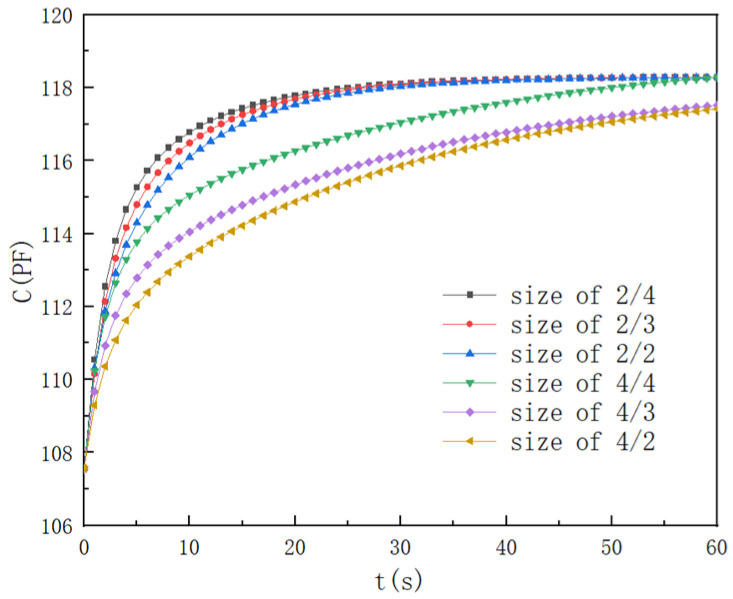
Upper electrode size effects.

To investigate the structural influence of the upper electrode on the sensor’s dynamic characteristics, the upper electrodes with bar width/gap widths of 2 μm/4 μm, 2 μm/3 μm, 3 μm/4 μm, 3 μm/2 μm, 4 μm/3 μm, and 4 μm/2 μm were selected. The other parameters were the same as before. Figure 5 presents the numerical results.

When the bar width was 2 μm and the gap width was 2 μm, the response times of *t*_30_, *t*_60_, and *t*_90_ were 1.32 s, 4.47 s, and 16.52 s, and as the gap widened from 2 μm to 3 μm, and to 4 μm again, the response times of *t*_60_ improved by over 15.4% and 15%, respectively. When the bar width was 4 μm and the gap width was 2 μm, the response times of *t*_30_, *t*_60_, and *t*_90_ were 2.56 s, 11.79 s, and 53.67 s, and as the gap was widened from 2 μm to 3 μm, and to 4 μm again, the response times of *t*_60_ improved by over 18.4% and 42%, respectively. Compared to the sensor with an upper electrode with a 4 μm bar, the response time *t*_90_ of the sensor with an upper electrode with a 2 μm bar improved by over 60% in the same gap width. The smaller grid width helped to shorten the diffusion distance of the water molecules, thereby accelerating the diffusion process of the water, and a larger gap width led to a greater film area exposed to the water molecules, which resulted in a faster response time.

## 3. Design and Fabrication of the Sensor

In this study, a parallel plate capacitive polymer humidity sensor with good dynamic characteristics, whose chip size was 3 mm × 3 mm and source area size was 2 mm × 2 mm, was the focus.

Among of single crystal Si, glass, ceramic, plastic and metal, single crystal Si slice is easier to mini-size and compatible with CMOS process. And thus, single crystal Si slice is elected as the substrate materials of the sensor. By oxidation process, SiO_2_ layer is produced as the insulation layer.

Al is used widely as a sensor electrode due to its cheaper, simpler technology and better stability, which is due to its outside oxide layer, Al_2_O_3_. Considering the stronger viscosity of an electrode with a SiO_2_ layer and down-lead, the metal Mo was used, and a two-layer structure of Al-Mo was adopted for the sensor electrode. The lower electrode was a slab, and the upper electrode was a grid to conveniently allow water molecules into or out of the film, whose bar width and gap width were both 2 μm.

**Figure 6:**
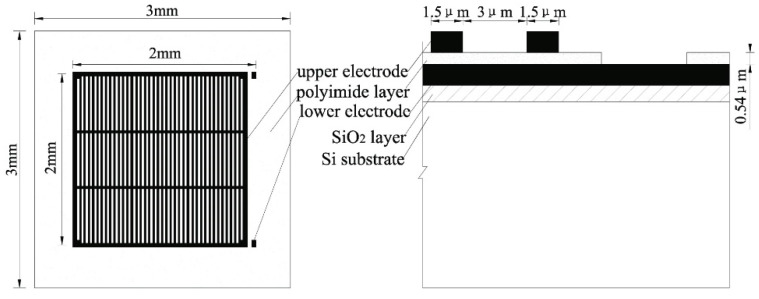
Sensor structure.

Among the various sensing moisture media, polymer material can be easily synthesized to be a film with hydrophobic groups as the skeleton and some hygroscopic molecules to avoid chemical absorption and agglomeration. Furthermore, polyimide has a good resistance to high temperature and radiation, is good for insulation and stability, is compatible with Si technology, etc. With these considerations, polyimide was selected. Figure 6 shows the sensor structure.

**Figure 7:**
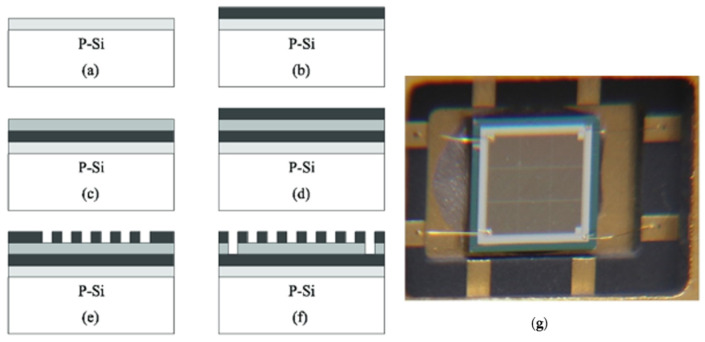
Fabrication process and the photo of the sensor.

The fabrication processes of the sensor are shown in Figure 7. Polyimide acid (PAA) was synthesized by our team, and its technology will be reported in another paper. In Figure 7a, the SiO_2_ layer is shown, which formed on the surface of a P-type single-crystal Si slice through oxygen. In Figure 7b, the lower electrode is shown plated with Mo and Al through evaporation. In Figure 7c, the sensing layer is shown, which was spun with polyimide acid (PAA), and then, the imidization of the sensing layer was performed. In Figure 7d, the upper electrode is shown plated with Al and Mo. In Figure 7e, the grid-shaped upper electrode is shown, which was formed through erosion. In Figure 7f, the down-lead hole of the lower electrode is shown eroding, and then, the devising, down-leading, and encapsulating of the sensors are shown. Figure 7g shows a photo of the sensor.

After trying many times, the imidization processes of polyimide film were adopted as follows: Firstly, the oven temperature was uniformly raised from the indoor temperature to 150 °C in 35 min and kept for 30 min. And then, the temperature was uniformly raised from 150 °C to 250 °C over 40 min and held for 30 min. Finally, the temperature was uniformly raised from 250 °C to 300 °C in 40 min and held for 60 min.

## 4. Performance of the Sensor

The sensor performance was as follows:

### 4.1. The Linearity, Sensitivity, and Hysteresis

The sensitivity (humidity coefficient) of the sensors is defined as the ratio of the capacitance change of the sensors to the relative humidity change under the condition of a constant temperature. The humidity hysteresis effect is defined as the humidity difference corresponding to the same capacitance between the absorption and adsorption processes. The linearity is described by the correlation coefficient *R*^2^ [15]:

(11) $$R^{2}={\frac{\left[\sum_{i=1}^{n}\left(x_{i}-\bar{x}\right)\left(C_{i}-\bar{C}\right)\right]}{\sum_{i=1}^{n}{\left(x_{i}-\bar{x}\right)}^{2}\sum_{i=1}^{n}{\left(C_{i}-\bar{C}\right)}^{2}}}^{2}$$

where *x_i_* is the humidity value of the *i*th test, $\bar{x}$ is the mean humidity value, *C_i_* is the *i*th capacitance tested corresponding to *x*_i_, and $\bar{C}$ is the mean capacitance value.

The static characteristics of the humidity sensor were measured by a system consisting of five saturation salt solutions of LiCl, MgCl_2_, NaBr, NaCl, and KNO_3_, separately [16], whose humidity range was between 12% RH and 92% RH. The system was located in a water tank with a constant temperature. The sensor capacitance was measured by a TH2619 high-speed capacitance meter from Tonghui Electronic Co., Ltd.(Shanghai, China), with a basic precision of 0.1%.

Prior to each test, the sensor was placed in a saturation solution of MgCl_2_ for about 1 h. In the test process, the sensor was put into a saturation solution of LiCl for about 50 min, firstly to achieve the balance state, and then, 10 capacitance values were read during the following 1 h. After this, the absorption test was performed with saturation solutions of MgCl_2_, NaBr, NaCl, and KNO_3_ in turn, according to the same methods as those used for LiCl. After that, the desorption test was performed immediately with saturation solutions of KNO_3_, NaCl, NaBr, MgCl_2_, and LiCl in turn.

**Figure 8:**
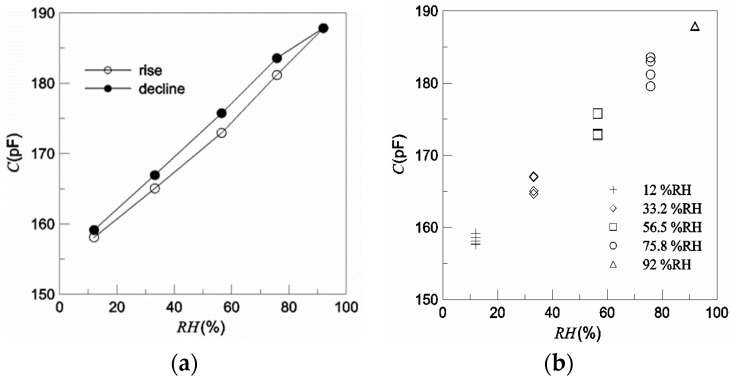
The test results. (**a**) Linearity, sensitivity, and hysteresis. (**b**) Repetitiveness.

Figure 8 shows the test results. The *R*^2^ of the sensor was 0.9965, the maximum hysteresis was 7.408% RH, the maximum sensitivity was 0.4264 pF/%RH from 56.5% RH to 75.8% RH, and the mean sensitivity was 0.3761 pF/%RH in the range of 0–100% RH. Due to some agglomeration or chemical absorption, the sensor capacitance in the dehumidifying process was larger than that in the humidifying process at the same humidity. The repeatability of a sensor is also a very important index for a good humidity sensor, and the maximum humidity deviation of the sensor was ±2.01% RH, which could have been caused by the nonuniform humidity of the test airflow and some condensation of water molecules in the film. The specific test process can be referred to in the literature [16].

### 4.2. The Response Time

**Figure 9:**
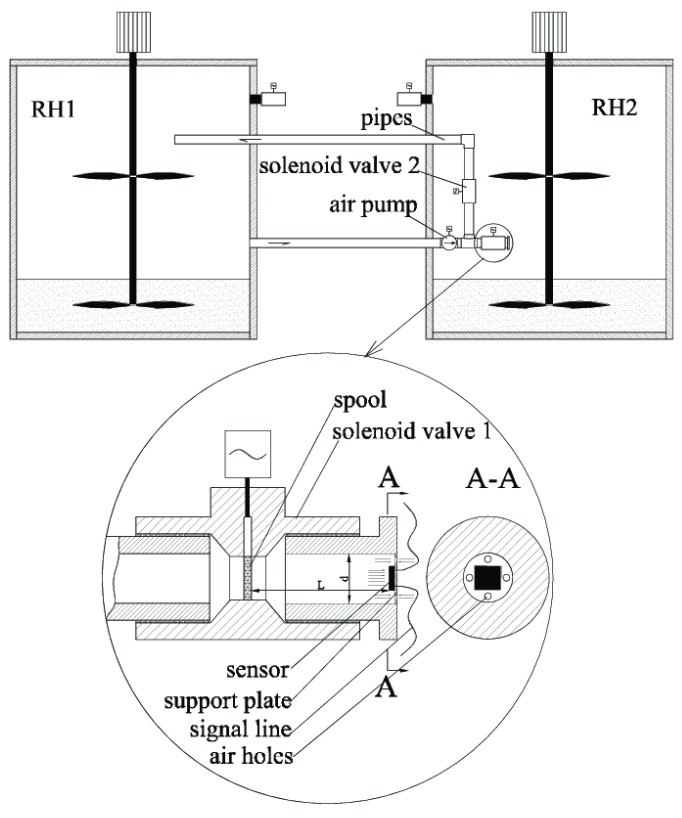
Facility for dynamic characteristics.

The test facility used for the transient response characteristics of the humidity sensors is shown schematically in Figure 9. It included generators, a pipeline system, and a measure and control system. The detailed testing procedure is referenced in [16]. Airflows with different humidity were provided by different humidity generators based on the saturation salt solution. In this test, the solutions of MgCl_2_ and NaCl were selected, whose corresponding relative humidity values were 33.2% RH and 75.8% RH at 25 °C, respectively. The relative humidity of the humidity generators was calibrated by a dew point meter with uncertainty less than ±0.5 °C from Sichuan Wande Gas Analysis Technology Development Co., Ltd.(Sichuan, China) The whole system was located in a constant-temperature water tank, whose temperature uncertainty was less than ±0.2 °C.

**Figure 10:**
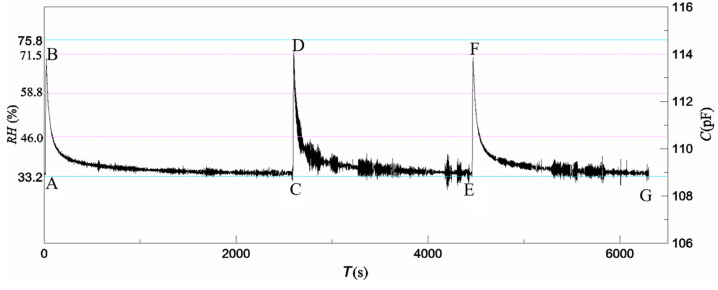
Dynamic characteristics of the sensor.

The sensor was placed in the test cell of the generator RH2. Prior to each test, the solenoid valve 1 was closed, and the air pump drove the air circulation of generator RH1 through the solenoid valve 2 until the balance state of the sensor was reached. When the test began, the solenoid valve 2 was closed, and solenoid valve 1 was opened at the same time by a programmer control. The air in the generator RH1 blew toward the sensor surface instead of the circulation. The sensor capacitance was read at TH2619 m at a frequency of 20 ms. The response time of the solenoid valves was less than 100 ms, and the pipe length from the switch point to the sensor surface was less than 10 cm. Three test results of the sensor are shown in Figure 10, of which the curves in A-B, C-D, and E-Fare the humidifying processes of blowing air with humidity RH1, and the curves inB-C, D-E, and F-Gare the naturally recovering processes from RH1 to RH2. The sensor displayed a typical adsorption time *t*_30_ of 1 s and a minimum adsorption time *t*_30_ of 850 ms over a range from 33.2% RH to 75.8% RH. This result is similar to the numerical result shown above in this paper. Several discrete points in Figure 10 resulted from an uneven humidity environment and pulsation of the air pump used in the test equipment.

### 4.3. The Stability

**Figure 11:**
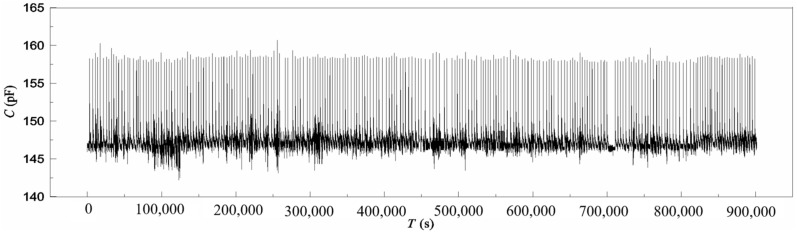
Stability of the sensor.

At 25 °C, the stability test of the sensor was performed by repeating the response time test mentioned above for 10 days. In each test, the airflow with 75.8% RH was blown onto the sensor surface for 10 s, and then, the desorption process lasted 1 h naturally until the balance state of the sensor in the environment of 33.2% RH was achieved. The same test was repeated constantly 230 times. Figure 11 shows the results of the test. The reason for the several discrete points in Figure 11 could be the same as detailed in Section 4.2.

Like the dynamic characteristics, at the instance the humidity changed in the test cell, the sensor capacitance increased rapidly, but the natural recovering processes were very slow. Among 230 curves, the curve configurations were almost the same, but there were some differences due to inappropriate operation and interference from outside. This result indicates that the sensor is stable.

A comparison of the sensing performance of various humidity sensors that have been reported in the following references is summarized in Table 3. It is clear that the humidity-sensitive element provided in this study performed better in terms of its fast detection of ambient humidity and the corresponding dynamic indices.

## 5. Conclusions

With the development of the measure and control fields, humidity sensors with good dynamic characteristics are needed. In this study, an effective numerical model and method for a parallel plate capacitance humidity sensor was built firstly, and then a sensor based on a Si substrate, Al-Mo electrodes, and a polyimide thin film was numerically predicted, fabricated, and tested. The numerical prediction proved valuable. The sensor developed in this study could be a promising candidate for use in quick humidity measurements. The linearity of the sensor was very good, the hysteresis was very small, and the sensitivity was high. The sensor displayed an average adsorption time of 1 s when the RH increased from 33.2% RH to 45.98% RH. The sensor demonstrated very good stability during a 240 h test at 25 °C.

## Figures and Tables

**Table 1:** Parameters used in the formulas.

Parameter	Value	Parameter	Value
humidity	33.2% RH~75.8% RH	A/m^2^	3.8 × 10^−7^
*γ_m_*	4.04%	δ/μm	0.75
*ε*_0_	8.85 × 10^−12^	*ε_p_*	2.9
*α*_0_	2.43 × 10^−3^	*ψ*_0_	0.836
*α*_1_	2.22 × 10^−4^	*β*_1_	4.9 × 10^−3^
*α*_2_	2.34 × 10^−5^	*β*_2_	−0.12
*B*/pa	101,325	*T*/°C	25

**Table 2:** Assessment results of grid independence.

Response Time (s)	*t*_30_	*t*_60_	*t*_90_
0.018	1.13	3.32	13.11
0.020	1.11	3.21	12.94
0.023	1.08	3.17	12.67

**Table 3:** Comparison of the sensing properties of various humidity sensors.

Literatures	Range	Response Time	Sensitivity	Moisture-Sensitive Film Materials
[11]	——	——	2.538 THz/RIU	Graphene/polysilicon/SiO_2_/Au
[12]	——	——	3.923 THz/RIU	Based on a flower-shaped graphene
[17]	18.48%RH–81.67%RH	13.3 s/12.4 s	——	10 nm thick silicon nitride film
[18]	10%RH–95%RH	All less than 1 s	——	Graphene oxide (GO) based ultra-thin and thin
[19]	11%RH–97%RH	20 s/12 s	369 pF/%RH	MoS_2_/GOQD
[20]	20%RH–90%RH	32 s/28 s	——	PVDF/PTPA nanomembrane
[21]	10%RH–60%RH	2 s/1.75 s	0.53 pF/%RH	Composed of MXene and single-walled carbon nanotubes (SWCNTs)
This work	33.2%RH–75.8%RH	1 s/850 ms	0.4264 pF/%RH	Polyimide film

## Data Availability

The original contributions presented in the study are included in the article, further inquiries can be directed to the corresponding author.
